# Optimizing the Agricultural Internet of Things (IoT) with Edge Computing and Low-Altitude Platform Stations

**DOI:** 10.3390/s24217094

**Published:** 2024-11-04

**Authors:** Deshan Yang, Jingwen Wu, Yixin He

**Affiliations:** 1 School of Mechanical Engineering, Tianjin University, Tianjin 300072, China; deshan.yang@zjxu.edu.cn; 2 College of Information Science and Engineering, Jiaxing University, Jiaxing 314001, China; 00193873@stu.zjxu.edu.cn; 3 Zhejiang Chengshi Robot Co., Ltd., Jiaxing 314001, China

**Keywords:** agricultural Internet of Things (IoT), edge computing, low-altitude platform stations (LAPSs), priority selection, total task processing delay

## Abstract

Using low-altitude platform stations (LAPSs) in the agricultural Internet of Things (IoT) enables the efficient and precise monitoring of vast and hard-to-reach areas, thereby enhancing crop management. By integrating edge computing servers into LAPSs, data can be processed directly at the edge in real time, significantly reducing latency and dependency on remote cloud servers. Motivated by these advancements, this paper explores the application of LAPSs and edge computing in the agricultural IoT. First, we introduce an LAPS-aided edge computing architecture for the agricultural IoT, in which each task is segmented into several interdependent subtasks for processing. Next, we formulate a total task processing delay minimization problem, taking into account constraints related to task dependency and priority, as well as equipment energy consumption. Then, by treating the task dependencies as directed acyclic graphs, a heuristic task processing algorithm with priority selection is developed to solve the formulated problem. Finally, the numerical results show that the proposed edge computing scheme outperforms state-of-the-art works and the local computing scheme in terms of the total task processing delay.

## 1. Introduction

In the agricultural Internet of Things (IoT), the adoption of low-altitude platform stations (LAPSs) significantly enhances the coverage, velocity of data acquisition, and connectivity reliability, particularly in regions with limited infrastructure [1]. LAPSs facilitate the efficient collection and transmission of data from IoT devices spread across agricultural land, enabling better crop management and production efficiency [2]. The integration of edge computing servers with LAPSs is instrumental in handling computationally intensive tasks, which can reduce the latency and bandwidth usage [3]. Additionally, processing data at the edge improves the task offloading performance of the agricultural IoT by minimizing long-distance data transmission, thereby increasing network resilience [4]. Therefore, in the agricultural IoT, the integration of LAPS with edge computing can optimize agricultural operations and simultaneously accelerate the implementation of Agriculture 4.0.

Agriculture 4.0 represents a transformative approach that leverages advanced techniques, such as big data and artificial intelligence, to enhance agricultural productivity and sustainability [5,6,7]. In particular, through the introduction of edge computing and LAPSs, real-time data processing becomes feasible, allowing for immediate responses to changing agricultural conditions [8,9,10]. This integration is critical to implementing the full potential of IoT in agriculture [11]. Therefore, several researchers have initiated studies to explore the implications of incorporating edge computing and LAPSs within the agricultural IoT. Specifically, to improve the average task processing ratio, the authors in [12] adopt the LAPS equipped with an edge computing server to provide computation offloading services. Inspired by this work, an optimal pre-offloading decision algorithm is proposed in [13]. Moreover, by taking into account offload decisions based on load balancing, the authors investigate the resource allocation problem for multi-user multi-server IoT applications in [14,15]. Under the constraints of queuing delays, the authors in [16] present an average task delay minimization scheme via jointly optimizing the LAPS deployment and device association. Furthermore, in the agricultural IoT with edge computing, dividing a task into multiple subtasks can enhance efficiency by allowing parallel processing, which speeds up the overall execution time [17]. Meanwhile, the failure of a single subtask does not necessarily compromise the entire operation. As discussed in [18,19], the authors utilize the features of deep neural networks to develop a task offloading scheme that facilitates the concurrent processing of multiple subtasks.

Although the mentioned solutions have significantly improved the task processing performance of the LAPS-aided agricultural IoT, there are still several issues that warrant further exploration.

- On the one hand, the works in [12,13] only consider the impact of a single edge computing server and fail to address the potential collaboration among multiple edge computing servers. This assumption limits the optimization of resources. Because a multi-server architecture can handle data tasks more efficiently, it can reduce latency and improve overall system performance. Therefore, we need to further investigate how multiple edge servers can work together to maximize the efficiency and responsiveness of agricultural data processing.
- On the other hand, although the works [14,15,16] explore task collaborative offloading using multiple edge computing servers installed on LAPSs, these studies are based on the implicit assumption that tasks to be offloaded can only be divided into two parts and cannot be further subdivided into multiple subtasks. In contrast, the authors in [17,18,19] focus on the offloading of multiple subtasks. Unfortunately, how to determine the priorities of subtasks to satisfy the practical task needs still requires further discussion.

To address the challenges mentioned above, this paper proposes an LAPS-aided edge computing architecture for the agricultural IoT, aimed at reducing the latency that occurs when data must be transferred to remote cloud servers for processing. Our main contributions are highlighted as follows.

- First, in the considered agricultural IoT scenario, each task is divided into multiple interdependent subtasks for processing. Next, we view the task dependencies as directed acyclic graphs (DAGs). Then, the problem of minimizing the total task processing delay is formulated by jointly considering task dependency and priority, equipment energy consumption, and the features of air–ground integrated communication.
- Second, to solve this non-convex optimization problem, we propose a heuristic task processing algorithm with priority selection by using graph theory. The proposed scheme ensures task dependency by facilitating collaboration among multiple edge computing servers.
- Finally, the numerical results verify that the proposed scheme significantly reduces the total task processing delay compared to state-of-the-art works [4,12,14] and the local computing scheme. In addition, the influence of device distribution on the total task processing delay is demonstrated.

## 2. System Model and Problem Formulation

### 2.1. LAPS-Aided Agricultural IoT Model

**Figure 1:**
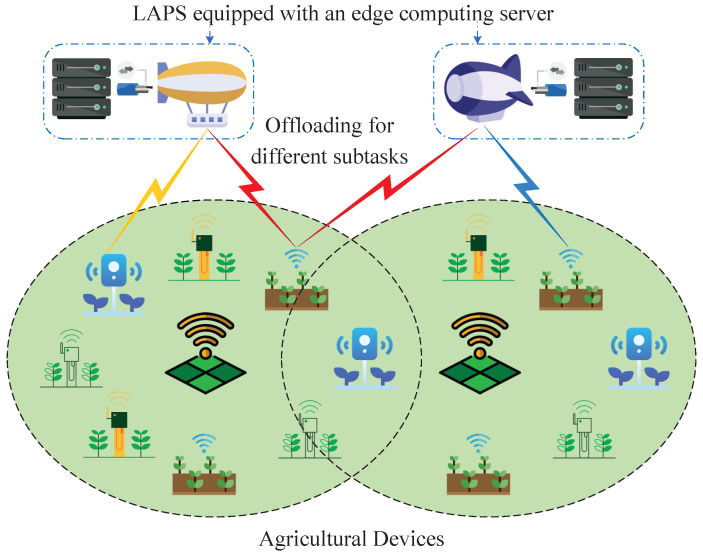
LAPS-aided agricultural IoT system.

Figure 1 depicts the considered LAPS-aided agricultural IoT system, consisting of *L* LAPSs and *D* agricultural devices. The sets of LAPSs and agricultural devices are defined as $L=\left\{1,...,L\right\}$ and $D=\left\{1,...,D\right\}$, respectively. Each LAPS is equipped with an edge computing server that has limited computational capacity. In this paper, LAPSs are uniformly distributed across the considered agricultural IoT scenarios. Meanwhile, LAPSs operate in a hovering mode to provide edge computing services. The tasks of the *d*-th $\left(\forall d\in D\right)$ device can be divided into *V* dependent subtasks (defined as $V=\left\{1,...,V\right\}$). In other words, in the considered scenario, we have *D* agricultural devices, each with a divisible and unprocessed task. Each unprocessed task can be segmented into *V* interdependent subtasks, which can be processed either at LAPSs or locally. The advantage of dividing a total task into several subtasks lies in enhanced efficiency and flexibility in processing. By segmenting tasks, the subtasks can be processed in parallel, thereby reducing the overall execution time. Furthermore, this approach allows for better resource allocation, as subtasks can be distributed across various computing nodes.

The processing of these tasks can be regarded as a DAG [20], where the node set $V_{d}=\left\{\left.v_{d}\right|\forall v\in V\right\}$ contains the *V* subtasks of the *d*-th device, and the directed edge set $E_{d}$ indicates the dependencies among the subtasks. The unprocessed subtasks are processed in the following provisions [4,21]. First, we determine the dependencies among the subtasks in advance. This is because some subtasks need to be started after other subtasks have been completed. Then, we prioritize each subtask according to its importance and urgency. Higher-priority tasks are handled in a timelier manner to ensure a prompt response. Additionally, the resource allocation must be taken into account, as some subtasks require specific computational capabilities. Finally, to ensure the availability of edge computing, the first and last subtasks must be processed locally.

**Figure 2:**
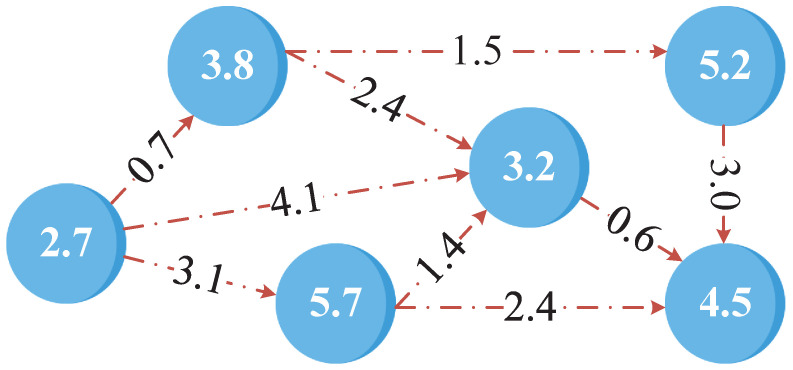
Subtask dependency based on the DAG.

As shown in Figure 2, the value marked on the $v_{d}$-th node represents the computational resources required by the corresponding subtask. Meanwhile, the values on the directed edges between two nodes indicate the data transmission costs between the subtasks. In the LAPS-aided agricultural IoT system, each subtask can be processed either on any edge computing server via air–ground integrated transmission or locally using the device’s own computational resources. Moreover, for the *d*-th device, its various subtasks can be offloaded to different edge computing servers for processing. Furthermore, due to dependencies among some subtasks, two dependent subtasks must be processed in a specific order and cannot be reversed.

### 2.2. Computing Model

This paper considers a non-preemptive task scheduling mechanism. Therefore, the execution of a subtask is not allowed to be interrupted until it is completed. In this situation, the task processing delay refers to the total time elapsed from the start of the first subtask to the completion of the last subtask. The *v*-th subtask of the *d*-th device can be processed either on an edge computing server or locally. For $v_{d}$, its task processing delay $\vartheta \left(d,v\right)$ is given by

(1) $$\begin{array}{c} \vartheta \left(d,v\right)=\min_{l\in \left\{L\cup \left\{d\right\}\right\}}\left\{\vartheta_{1}\left(d,v,l\right)+\vartheta_{2}\left(d,v,l\right)\right\}, \end{array}$$

where $l\in \left\{L\cup \left\{d\right\}\right\}$ represents the corresponding computing server; $\vartheta_{1}\left(d,v,l\right)$ is the actual earliest start time when the *v*-th subtask of the *d*-th device is offloaded the corresponding computing server *l*; and $\vartheta_{2}\left(d,v,l\right)$ is the transmission and processing delay of the *v*-th subtask on the corresponding computing server *l*.

Since subtasks need to be processed in order, $\vartheta_{1}\left(d,v,l\right)$ can be further rewritten as

(2) $$\begin{array}{c} \vartheta_{1}\left(d,v,l\right)=\max\left\{\kappa \left(d,v,l\right),{\tilde{\vartheta }}_{1}\left(d,v,l\right)\right\}, \end{array}$$

where $\kappa \left(d,v,l\right)$ is the theoretical earliest start time of the *v*-th subtask of the *d*-th device on the corresponding computing server *l* and ${\tilde{\vartheta }}_{1}\left(d,v,l\right)$ is the earliest time that the corresponding computing server *l* is ready to process the *v*-th subtask of the *d*-th device. By recursion, ${\tilde{\vartheta }}_{1}\left(d,v,l\right)$ can be further represented as

(3) $$\begin{array}{c} {\tilde{\vartheta }}_{1}\left(d,v,l\right)=\max_{v^{\prime }\in \mathrm{Pre}\left\{v\right\}}\left\{\vartheta \left(d,v^{\prime }\right)+T_{l,l^{\prime }}\left(v,v^{\prime }\right)\right\}, \end{array}$$

where $\mathrm{Pre}\left\{v\right\}$ is the set of immediate predecessor subtasks of the *v*-th subtask of the *d*-th device. For the first subtask (i.e., v=1), we have ${\tilde{\vartheta }}_{1}\left(d,1,l\right)=0$. When $v^{\prime }$ is processed on $l^{\prime }$ and *v* is processed on *l*, the information exchange time $T_{l,l^{\prime }}\left(v,v^{\prime }\right)$ between subtasks *v* and $v^{\prime }$ is

(4) $$\begin{array}{c} T_{l,l^{\prime }}\left(v,v^{\prime }\right)=\frac{I\left(v,v^{\prime }\right)}{C\left(l,l^{\prime }\right)}, \end{array}$$

where $I\left(v,v^{\prime }\right)$ is the amount of information exchange between subtasks *v* and $v^{\prime }$; $C\left(l,l^{\prime }\right)$ is the channel capacity between servers *l* and $l^{\prime }$. Note that because subtasks have dependencies, information needs to be exchanged after subtasks *v* and $v^{\prime }$ are processed. In this situation, $T_{l,l^{\prime }}\left(v,v^{\prime }\right)$ is the delay for exchanging this information.

### 2.3. Transmission Model

According to [22], in the air–ground channel, communication links can be divided into two parts: line-of-sight (LoS) and non-line-of-sight (NLoS) components. Therefore, the path loss $\mathrm{PL}\left(l,\;d\right)$ between the *l*-th edge computing server installed on the LAPS and the *d*-th device can be expressed as

(5) $$\begin{array}{c} \mathrm{PL}\left(l,\;d\right)=P_{l,d}^{\mathrm{LoS}}\times {\mathrm{PL}}_{l,d}^{\mathrm{LoS}}+P_{l,d}^{\mathrm{NLoS}}\times {\mathrm{PL}}_{l,d}^{\mathrm{NLoS}}. \end{array}$$

In (5), $P_{l,d}^{\mathrm{LoS}}$ and $P_{l,d}^{\mathrm{NLoS}}$ are the probabilities of occurrence of LoS and NLoS components, respectively. We can obtain

(6) $$\begin{array}{c} P_{l,d}^{\mathrm{LoS}}=\frac{1}{1+\omega_{1}\exp\left\{\omega_{2}\left[\frac{180}{\pi }\arctan\left(\frac{h_{l}}{\psi_{l,d}}\right)-\omega_{1}\right]\right\}}, \end{array}$$

where $\omega_{1}$ and $\omega_{2}$ are *S*-band constants that depend on the environment; $h_{l}$ is the height of the *l*-th LAPS; $\psi_{l,d}$ is the horizontal plane projection distance between the *l*-th LAPS and the *d*-th device. Similarly, $P_{l,d}^{\mathrm{NLoS}}$ is given by

(7) $$\begin{array}{c} P_{l,d}^{\mathrm{NLoS}}=\frac{\omega_{1}\exp\left\{\omega_{2}\left[\frac{180}{\pi }\arctan\left(\frac{h_{l}}{\psi_{l,d}}\right)-\omega_{1}\right]\right\}}{1+\omega_{1}\exp\left\{\omega_{2}\left[\frac{180}{\pi }\arctan\left(\frac{h_{l}}{\psi_{l,d}}\right)-\omega_{1}\right]\right\}}. \end{array}$$

Additionally, ${\mathrm{PL}}_{l,d}^{\mathrm{LoS}}$ and ${\mathrm{PL}}_{l,d}^{\mathrm{NLoS}}$ represent the loss values (in dB) corresponding to the LoS and NLoS components, respectively, denoted as

(8) $$\begin{array}{c} {\mathrm{PL}}_{l,d}^{\mathrm{LoS}}=\chi_{\mathrm{LoS}}{\left(\frac{4\pi f_{c}\sqrt{{\left(\psi_{l,d}\right)}^{2}+{\left(h_{l}\right)}^{2}}}{c}\right)}^{2}, \end{array}$$

and

(9) $$\begin{array}{c} {\mathrm{PL}}_{l,d}^{\mathrm{NLoS}}=\chi_{\mathrm{NLoS}}{\left(\frac{4\pi f_{c}\sqrt{{\left(\psi_{l,d}\right)}^{2}+{\left(h_{l}\right)}^{2}}}{c}\right)}^{2}, \end{array}$$

where $f_{c}$ is the carrier frequency; *c* is the velocity of light; and $\chi_{\mathrm{LoS}}$ and $\chi_{\mathrm{NLoS}}$ are the additional path loss values for the LoS and NLoS components, respectively. Furthermore, in order to avoid co-channel interference, the orthogonal frequency division multiple access (OFDMA) technique is adopted, and the Hungarian algorithm is used to pre-allocate the spectrum [23].

### 2.4. Energy Consumption Model

In the LAPS-aided agricultural IoT, the total energy consumption $E_{\mathrm{tot}}$ for task offloading can be calculated as

(10) $$\begin{array}{c} E_{\mathrm{tot}}=E_{\mathrm{trans}}+E_{\mathrm{com}}, \end{array}$$

where $E_{\mathrm{trans}}$ is the energy consumption required for task transmission and $E_{\mathrm{com}}$ is the energy consumption required for task computing. Specifically, $E_{\mathrm{com}}$ can be calculated as

(11) $$\begin{array}{c} E_{\mathrm{com}}=\sum_{d=1}^{D}\sum_{v=1}^{\left|V_{d}\right|}\zeta_{\left\{l_{d}^{v}=d\right\}}\Phi_{d}\vartheta_{2}\left(d,v,d\right), \end{array}$$

where $\left|V_{d}\right|$ is the last subtask of the *d*-th device; $\Phi_{d}$ is the energy consumption required for computing tasks by the *d*-th device per unit time; $l_{d}^{v}$ is the computing server selected by the *v*-th subtask of the *d*-th device; and $\zeta_{\left\{\cdot \right\}}$ is the binary indicator variable, $\zeta_{\left\{\cdot \right\}}\in \left\{0,\;1\right\}$. We have $\zeta_{\left\{l_{d}^{v}=d\right\}}\;=\;1$ and $\zeta_{\left\{l_{d}^{v}\neq d\right\}}\;=\;0$.

In addition, $E_{\mathrm{trans}}$ can be calculated as

(12) $$\begin{array}{ccc} E_{\mathrm{trans}}\; & = & \;\sum_{d=1}^{D}\sum_{v=1}^{\left|V_{d}\right|}P_{d}\left(\frac{\zeta_{\left\{l_{d}^{v}>0\right\}}S\left(d,v\right)}{C\left(d,l_{d}^{v}\right)}\right)\\ \; & & \;+\sum_{d=1}^{D}\sum_{v=1}^{\left|V_{d}\right|}P_{d}\left(\sum_{v^{\prime }\in \mathrm{Pre}\left\{v\right\}}\zeta_{\left\{l_{d}^{v}l_{d}^{v^{\prime }}<0\right\}}T_{a,a^{\prime }}\left(v,v^{\prime }\right)\right), \end{array}$$

where $P_{d}$ is the transmitted power of the *d*-th device; $S\left(d,v\right)$ is the size of the *v*-th subtask of the *d*-th device; $C\left(d,l_{d}^{v}\right)$ is the channel capacity between the *d*-th device and computing server $l_{d}^{v}$.

### 2.5. Total Task Processing Delay Minimization Problem

By jointly considering the task dependency, priority, equipment energy consumption, and the characteristics of air–ground integrated communication, a mathematical formulation is presented for minimizing the total task processing delay problem.

(13a) $$\begin{array}{cc} \max_{A,\;B\;}\; & \;\sum_{d=1}^{D}\vartheta \left(d,\left|V_{d}\right|\right) \end{array}$$

(13b) $$\begin{array}{cc} s.t.\; & \;E_{\mathrm{tot}}\leq E_{\mathrm{th}}, \end{array}$$

(13c) $$\begin{array}{cc} \; & \;l_{d}^{1}=l_{d}^{\left|V_{d}\right|}=d, \end{array}$$

where $E_{\mathrm{th}}$ is the energy consumption threshold; $A=\left\{\left.l_{d}^{v}\right|\forall d\in D,\;\forall v\in V_{d}\right\}$, and A is the set of servers; and B is the subtask processing sequence set, $B=\left\{\left.b_{d}^{v}\right|\forall d\in D,\;\forall v\in V_{d}\right\}$. Given $l_{d}^{v}=l_{d}^{v^{\prime }}$, $b_{d}^{v}<b_{d}^{v^{\prime }}$ indicates that the server processes subtask *v* first, followed by subtask $v^{\prime }$. Additionally, constraint (13b) ensures that energy consumption cannot exceed the threshold $E_{\mathrm{th}}$. Constraint (13c) restricts the first and last subtasks to be processed locally. In this paper, we minimize the sum of task processing delays and thus the average task processing delay per device (minimizing the sum is essentially minimizing the average). This optimization objective can be regarded as a baseline to measure the task processing performance of the agricultural IoT [12,24].

## 3. Task Processing Scheme with Priority Selection

Since the formulated problem in (13) involves mixed-integer nonlinear programming, it is challenging to solve it directly. Therefore, following the approach described in [4], this paper designs a heuristic algorithm by using DAGs. Theoretically, deep reinforcement learning approaches can be used to deal with the formulated total task processing delay minimization problem. However, this paper develops a heuristic algorithm (see Algorithm 1 below for detailed steps) instead of a deep reinforcement learning approach for the following reasons. On the one hand, the heuristic algorithm can take advantage of the characteristics of DAGs and design specific heuristic functions to quickly find efficient solutions. In contrast, deep reinforcement learning approaches require a large amount of training data and iteration, and may be less efficient than heuristic algorithms. On the other hand, heuristic algorithms typically exhibit high interpretability, making their procedural steps and decision-making processes more accessible for understanding and analysis. In contrast, deep learning models are frequently regarded as ’black boxes’ that face challenges in elucidating their decision-making mechanisms. Specifically, to ensure dependency, the subtasks are arranged in sequence, and their priorities are subsequently sorted. For the *v*-th subtask of the *d*-th device, the sorting level $\mathrm{Level}\left(d,v\right)$ can be represented as

(14) $$\begin{array}{ccc} \mathrm{Level}\left(d,v\right)\; & = & \;\max_{v^{\prime }\in \mathrm{Seq}\left\{v\right\}}\left\{\mathrm{Level}\left(d,v^{\prime }\right)+\tilde{T_{1}}\left(v,v^{\prime }\right)\right\}\\ \; & & \;+\tilde{T_{2}}\left(d,v\right), \end{array}$$

where $\mathrm{Seq}\left\{v\right\}$ is the set of subtasks to be processed after the *v*-th subtask; $\tilde{T_{1}}\left(v,v^{\prime }\right)$ is the average information exchange time between the *v*-th subtask and its subsequent $v^{\prime }$-th subtask; and $\tilde{T_{2}}\left(d,v\right)$ is the average delay required to process the *v*-th subtask. In (14), $\tilde{T_{1}}\left(v,v^{\prime }\right)$ can be further rewritten as

(15) $$\begin{array}{c} \tilde{T_{1}}\left(v,v^{\prime }\right)=\frac{\left(L+1\right)I\left(v,v^{\prime }\right)}{\sum_{l=1}^{L}C\left(d,l\right)+\frac{1}{2}\sum_{l=1}^{L}\sum_{l^{\prime }\neq l}C\left(l,l^{\prime }\right)}, \end{array}$$

where $C\left(d,l\right)$ is the channel capacity between the *d*-th device and *l*-th LAPS. Similarly, $\tilde{T_{2}}\left(d,v\right)$ can be further expressed as

(16) $$\begin{array}{c} \tilde{T_{2}}\left(d,v\right)=\frac{\vartheta_{2}\left(d,v,d\right)+\sum_{l+1}^{L}\vartheta_{2}\left(d,v,l\right)}{L+1}. \end{array}$$

According to (14)–(16), we have $\mathrm{Level}\left(d,v\right)>\mathrm{Level}\left(d,v^{\prime }\right)$. If $\mathrm{Level}\left(d,v\right)<\mathrm{Level}\left(d,v^{\prime }\right)$, dependencies between subtasks cannot be guaranteed [25,26,27].
**Algorithm 1** Task processing algorithm with priority selection1:**Initialization**2:     Set *L*, *D*, *V*, $S\left(d,v\right)$, and other parameters related to the LAPS-aided agricultural IoT.3:     The Hungarian algorithm is used to pre-allocate the spectrum.4:     According to (14), we calculate $\mathrm{Level}\left(d,v\right)$ for ∀l∈L and ∀d∈D.5:**repeat**6:     In each $G_{d}$, we select the unprocessed subtask with the highest $\mathrm{Level}\left(d,v\right)$.7:     The selected subtasks form the candidate set for processing.8:     We select a specific subtask with highest $\Theta \left(d,v\right)$ from the candidate set by using (17).9:     According to (1), we calculate $\vartheta \left(d,v\right)$.10:   This specific subtask with highest $\Theta \left(d,v\right)$ is assigned to the corresponding server (the *l*-th LAPS or the *d*-th device) with lowest $\vartheta \left(d,v\right)$.11:   The corresponding server processes this subtask and marks it as processed.12:**until** All subtasks have been processed13:Output the optimal task offloading policy for the LAPS-aided agricultural IoT.

To optimize the processing of subtasks that involve high computational costs and energy consumption, while simultaneously minimizing the total task processing delay and reducing the energy consumption of agricultural equipment, the priority $\Theta \left(d,v\right)$ of the subtasks can be defined as

(17) $$\begin{array}{ccc} \Theta \left(d,v\right)\; & = & \;\varpi_{d}^{1}\mathrm{Level}\left(d,v\right)+\varpi_{d}^{2}\Phi_{d}\vartheta_{2}\left(d,v,d\right)\\ \; & & \;-\frac{L\varpi_{d}^{2}P_{d}S\left(d,v\right)}{\sum_{l=1}^{L}C\left(d,l\right)}, \end{array}$$

where $\varpi_{d}^{1}$ and $\varpi_{d}^{2}$ are the weight coefficients, corresponding to the computational cost and energy consumption of the *d*-th device, respectively. In particular, $\varpi_{d}^{1}=1-\varpi_{d}^{2}$.

In summary, the task processing algorithm with priority selection is outlined in Algorithm 1. First, we set the simulation parameters for the LAPS-aided agricultural IoT system and allocate spectrum resources by using the Hungarian algorithm. Following this, we employ (14) to compute $\mathrm{Level}\left(d,v\right)$, which assists in sorting all subtasks. Additionally, the DAG is used to map out the dependencies among the subtasks. In particular, to satisfy constraint (13c), we need to ensure that the first and last subtasks are processed locally. Then, based on the highest $\mathrm{Level}\left(d,v\right)$, we can generate a candidate set from which subtasks are selected for execution on the appropriate computing server. The selection of servers should consider $\Theta \left(d,v\right)$ and $\vartheta \left(d,v\right)$ jointly. Finally, the procedure continues iteratively until all subtasks are processed.

Discussion: Due to the potential existence of multiple local optima in the formulated problem, conventional optimization methods often encounter challenges in obtaining global optima within a reasonable timeframe. Motivated by the above, we propose a heuristic task processing algorithm that effectively explores the solution space to identify near-global optima or satisfactory local optima within acceptable computational times. Furthermore, heuristic edge computing solutions allocate data processing and analysis tasks near the network edge, thereby reducing the load on central servers and improving the system responsiveness. This significantly enhances the overall performance and efficiency of the LAPS-aided agricultural IoT.

## 4. Simulation Results

In this section, we evaluate the task processing performance of the LAPS-aided agricultural IoT through simulations. Specifically, in the LAPS-aided agricultural IoT, *D* devices are uniformly distributed. The key simulation parameters are set to $L=\left[2,\;5,\;8,\;10\right]$, $D=\left[20,\;200\right]$, V=55, $P_{d}=23$ dBm, $h_{l}=300$ m, $\omega_{1}=9.61$, $\omega_{2}=0.16$, and $S\left(d,v\right)=\left[300,\;1000\right]$ KB. As discussed in [4], the number of CPU cycles required for each sub-task is $\left[0.1,\;0.2\right]$ GHz. The number of CPU cycles per local device is 0.3 GHz, and each edge computing server installed on the LAPS has 6 GHz of CPU cycles. Additionally, the simulation results are expressed as the average values after $10^{4}$ iterations. This paper compares the proposed solution with four different schemes, namely Scheme 1 [12], Scheme 2 [4], Scheme 3 [14], and Scheme 4. Specifically, Scheme 1 employs an air–ground integrated architecture to handle tasks. In this architecture, a single LAPS is utilized to provide task offloading services. Second, Scheme 2 adopts a multi-server architecture to handle multiple interdependent subtasks. Third, Scheme 3 also utilizes a multi-server architecture, incorporating load balancing considerations. Finally, Scheme 4 represents a local computing architecture, which serves as a comparative benchmark.

**Figure 3:**
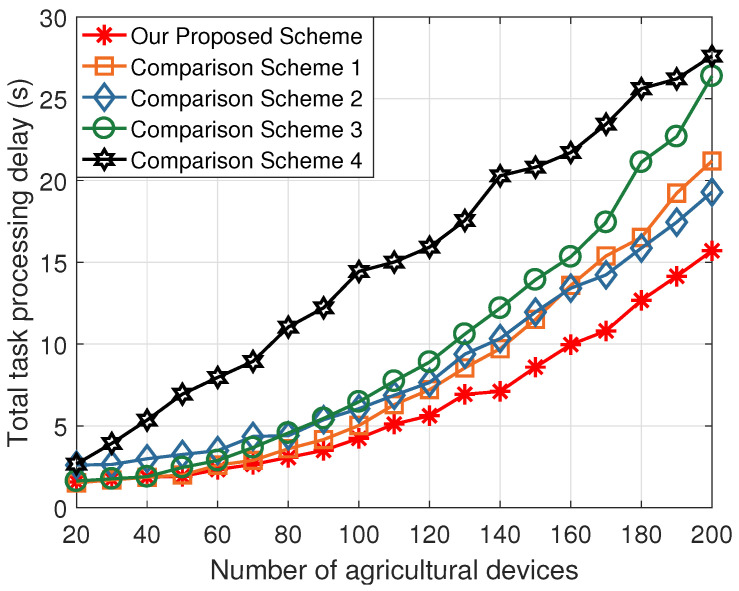
The total task processing delay versus the number of agricultural devices.

Figure 3 shows the curve of total task processing delay across various schemes as the number of agricultural devices changes. Notably, compared to state-of-the-art works and the local computing scheme, the proposed task offloading scheme exhibits the lowest total task processing delay. This is because Scheme 1 employs only a single LAPS as the edge computing server and does not divide the task into multiple subtasks. Moreover, Schemes 2 and 3 neglect the performance gains brought by air–ground integrated communication. Furthermore, Scheme 4 exhibits the highest total task processing delay, primarily due to the limited computing capabilities of the agricultural devices. Therefore, the proposed task offloading scheme can offer benefits to the agricultural IoT, enhancing the productivity and sustainability in farming practices.

**Figure 4:**
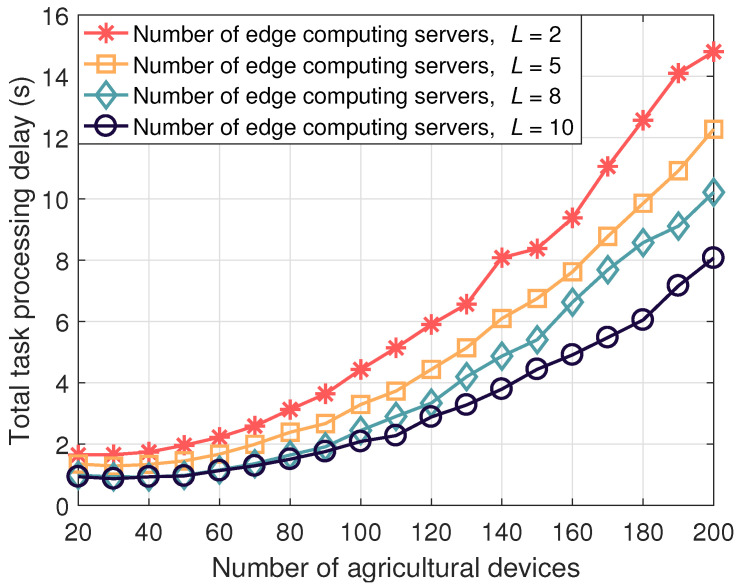
LAPS-aided agricultural IoT system.

Additionally, Figure 4 illustrates the impact of the number of edge computing servers on the total task processing delay. It is evident that increasing the number of edge computing servers can further reduce the total task processing delay. The reason for this is that the LAPS-aided agricultural IoT has access to more computational resources for task processing. However, an increase in the number of servers also leads to increased algorithmic complexity. Thus, optimizing the number and deployment of servers represents an interesting direction for further research.

**Figure 5:**
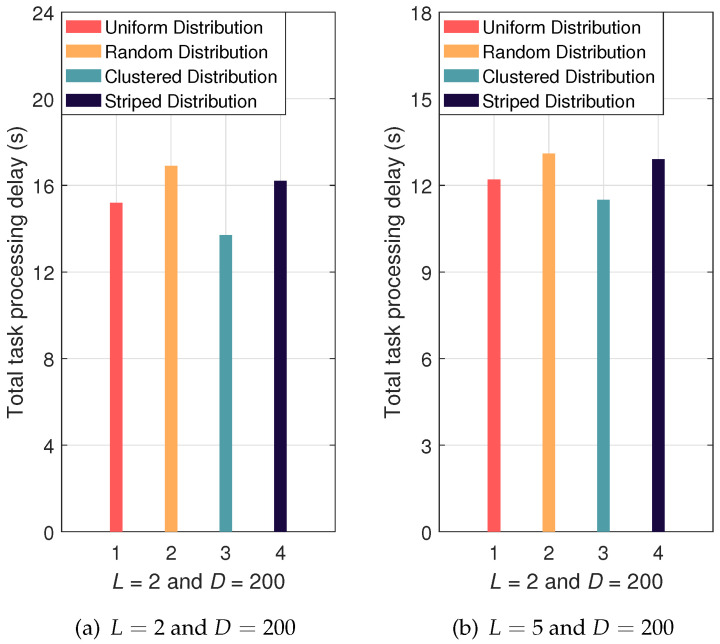
The impact of the device distributions on the total task processing delay.

Finally, Figure 5a,b give the impact of device distributions on the task processing performance of LAPS-aided agricultural IoT. It is observed that different distributions lead to varying total task processing delays. Compared to the uniform distribution, the clustered distribution exhibits lower total task processing delay. This is because edge computing servers can be effectively deployed within clusters to enhance offloading performance. As discussed above, optimizing device distribution can further reduce the total task processing delay and enhance the task processing capacity of agricultural IoT systems.

## 5. Conclusions

In this paper, we investigated the task offloading problem in the LAPS-aided agricultural IoT. First, edge computing servers were installed on LAPSs to address the problem of limited computing capacity of agricultural devices. Then, the task was divided into multiple subtasks, with dependencies between them established using DAGs. Subsequently, we formulated the problem of minimizing the total task processing delay, which was constrained by task dependencies and priorities, as well as equipment energy consumption. Considering the non-convex nature of the problem, a heuristic task processing algorithm was developed, incorporating priority selection. Finally, the numerical results demonstrated that the proposed task offloading scheme significantly reduced the total task processing delay compared to state-of-the-art works and the local computing scheme. As future work, we plan to optimize the distribution of these LAPSs and incorporate their mobility into the problem formulation. This approach aims to further enhance the performance of edge computing services in agricultural IoT applications, ensuring more efficient data processing and resource management in dynamic agricultural environments.

## Data Availability

Data are contained within the article.

## References

[1] Fu R., Ren X., Li Y., Wu Y., Sun H., Al-Absi M.A. (2023). Machine-learning-based UAV-assisted agricultural information security architecture and intrusion detection. IEEE Internet Things J..

[2] Adil M., Jan M.A., Liu Y., Abulkasim H., Farouk A., Song H. (2023). A systematic survey: Se-curity threats to UAV-aided IoT applications, taxonomy, current challenges and requirements with future research directions. IEEE Trans. Intell. Transp. Syst..

[3] Bai Z., Lin Y., Cao Y., Wang W. (2024). Delay-aware cooperative task offloading for multi-UAV enabled edge-cloud computing. IEEE Trans. Mob. Comput..

[4] Han Y., Zhao Z., Mo J., Shu C., Min G. Efficient task offloading with dependency guar-antees in ultra-dense edge networks. Proceedings of the IEEE Global Communications Conference (GLOBECOM).

[5] Zheng K., Jiang G., Liu X., Chi K., Yao X., Liu J. (2023). DRL-based offloading for computation delay minimization in wireless-powered multi-access edge computing. IEEE Trans. Commun..

[6] He Y., Huang F., Wang D., Zhang R., Gu X., Pan J. (2024). NOMA-enhanced cooperative re-laying systems in drone-enabled IoV: Capacity analysis and height optimization. IEEE Trans. Veh. Technol..

[7] Xiong S., Wang Z., Ni Q., Han X. (2024). PoMC: An efficient blockchain consensus mechanism for agricultural Internet of Things. IEEE Internet Things J..

[8] Li X., Hou B., Zhang R., Liu Y. (2023). A review of RGB image-based Internet of Things in smart agriculture. IEEE Sens. J..

[9] He Y., Wang D., Huang F., Zhang R., Min L. (2024). Aerial-ground integrated vehicular networks: A UAV-vehicle collaboration perspective. IEEE Trans. Intell. Transp. Syst..

[10] Adil M., Abulkasim H., Farouk A., Song H. (2024). R3ACWU: A lightweight, trustworthy au-thentication scheme for UAV-assisted IoT applications. IEEE Trans. Intell. Transp. Syst..

[11] Rashid L., Rubab S., Alhaisoni M., Alqahtani A., Alsubai S., Binbusayyis A., Bukhari S.A.C. (2022). Analysis of dimensionality reduction techniques on Internet of Things data using machine learning. Sus. Energy Technol. Assess.

[12] He Y., Wang D., Huang F., Zhang R. (2022). An MEC-enabled framework for task offloading and power allocation in NOMA enhanced ABS-assisted VANETs. IEEE Commun. Lett..

[13] Qian P., Wang L., Lin Y., Du J., Dong X. (2023). Joint power allocation and task offloading in NOMA enhanced MEC for ABS-assisted ITS. IEEE Commun. Lett..

[14] Dai Y., Xu D., Maharjan S., Zhang Y. (2019). Joint load balancing and offloading in vehicular edge computing and networks. IEEE Internet Things J..

[15] Duan S., Lyu F., Wu H., Chen W., Lu H., Dong Z., Shen X. (2024). MOTO: Mobility-aware online task offloading with adaptive load balancing in small-cell MEC. IEEE Trans. Mob. Comput..

[16] Han Z., Zhou T., Xu T., Hu H. (2023). Joint user association and deployment optimization for delay-minimized UAV-aided MEC networks. IEEE Wirel. Commun. Lett..

[17] Shu C., Zhao Z., Han Y., Min G., Duan H. (2020). Multi-user offloading for edge computing networks: A dependency-aware and latency-optimal approach. IEEE Internet Things J..

[18] Gao M., Shen R., Shi L., Qi W., Li J., Li Y. (2023). Task partitioning and offloading in DNN-task enabled mobile edge computing networks. IEEE Trans. Mob. Comput..

[19] Xu X., Yan K., Han S., Wang B., Tao X., Zhang P. (2024). Learning-based edge-device collab-orative DNN inference in IoVT networks. IEEE Internet Things J..

[20] Trinh B., Muntean G.-M. (2023). A deep reinforcement learning-based offloading scheme for multi-access edge computing-supported extended reality systems. IEEE Trans. Veh. Technol..

[21] Liu S., Yu Y., Lian X., Feng Y., She C., Yeoh P.L., Guo L., Vucetic B., Li Y. (2023). Dependent task scheduling and offloading for minimizing deadline violation ratio in mobile edge computing networks. IEEE J. Sel. Areas Commun..

[22] Bai Y., Zhao H., Zhang X., Chang Z., Jäntti R., Yang K. (2023). Toward autonomous multi-UAV wireless network: A survey of reinforcement learning-based approaches. IEEE Commun. Surv. Tuts..

[23] Alam M.Z., Jamalipour A. (2022). Multi-agent DRL-based Hungarian algorithm (MADRLHA) for task offloading in multi-access edge computing Internet of Vehicles (IoVs). IEEE Trans. Wirel. Commun..

[24] Taleb T., Samdanis K., Mada B., Flinck H., Dutta S., Sabella D. (2017). On multi-access edge computing: A survey of the emerging 5G network edge cloud architecture and orchestration. IEEE Commun. Surv. Tuts..

[25] Zhao M., Zhang X., He Z., Chen Y., Zhang Y. (2024). Dependency-aware task scheduling and layer loading for mobile edge computing networks. IEEE Internet Things J..

[26] Tang Z., Lou J., Jia W. (2023). Layer dependency-aware learning scheduling algorithms for containers in mobile edge computing. IEEE Trans. Mob. Comput..

[27] Xu B., Kuang Z., Gao J., Zhao L., Wu C. (2023). Joint offloading decision and trajectory design for UAV-enabled edge computing with task dependency. IEEE Trans. Wirel. Commun..

